# Endocrine and metabolic factors and the risk of idiopathic pulmonary fibrosis: a Mendelian randomization study

**DOI:** 10.3389/fendo.2023.1321576

**Published:** 2024-01-08

**Authors:** Yan Jiang, Rumeng Chen, Shuling Xu, Yining Ding, Mengling Zhang, Meihua Bao, Binsheng He, Sen Li

**Affiliations:** ^1^ School of Basic Medicine, Changsha Medical University, Changsha, China; ^2^ The Hunan Provincial Key Laboratory of the TCM Agricultural Biogenomics, Changsha Medical University, Changsha, China; ^3^ Hunan Key Laboratory of the Research and Development of Novel Pharmaceutical Preparations, School of Pharmaceutical Science, Changsha Medical University, Changsha, China; ^4^ School of Life Sciences, Beijing University of Chinese Medicine, Beijing, China; ^5^ School of Stomatology, Changsha Medical University, Changsha, China

**Keywords:** endocrine factors, metabolic factors, idiopathic pulmonary fibrosis, Mendelian randomization, UK Biobank

## Abstract

**Background:**

Previous observational studies have investigated the association between endocrine and metabolic factors and idiopathic pulmonary fibrosis (IPF), yet have produced inconsistent results. Therefore, it is imperative to employ the Mendelian randomization (MR) analysis method to conduct a more comprehensive investigation into the impact of endocrine and metabolic factors on IPF.

**Methods:**

The instrumental variables (IVs) for 53 endocrine and metabolic factors were sourced from publicly accessible genome-wide association study (GWAS) databases, with GWAS summary statistics pertaining to IPF employed as the dependent variables. Causal inference analysis encompassed the utilization of three methods: inverse-variance weighted (IVW), weighted median (WM), and MR-Egger. Sensitivity analysis incorporated the implementation of MR-PRESSO and leave-one-out techniques to identify potential pleiotropy and outliers. The presence of horizontal pleiotropy and heterogeneity was evaluated through the MR-Egger intercept and Cochran’s Q statistic, respectively.

**Results:**

The IVW method results reveal correlations between 11 traits and IPF. After correcting for multiple comparisons, seven traits remain statistically significant. These factors include: “Weight” (OR= 1.44; 95% CI: 1.16, 1.78; *P*=8.71×10^-4^), “Body mass index (BMI)” (OR= 1.35; 95% CI: 1.13, 1.62; *P*=1×10^-3^), “Whole body fat mass” (OR= 1.40; 95% CI: 1.14, 1.74; *P*=1.72×10^-3^), “Waist circumference (WC)” (OR= 1.54; 95% CI: 1.16, 2.05; *P*=3.08×10^-3^), “Trunk fat mass (TFM)” (OR=1.35; 95% CI: 1.10,1.65; *P*=3.45×10^-3^), “Body fat percentage (BFP)” (OR= 1.55; 95% CI: 1.15,2.08; *P*=3.86×10^-3^), “Apoliprotein B (ApoB)” (OR= 0.78; 95% CI: 0.65,0.93; *P*=5.47×10^-3^). Additionally, the sensitivity analysis results confirmed the reliability of the MR results.

**Conclusion:**

The present study identified causal relationships between seven traits and IPF. Specifically, ApoB exhibited a negative impact on IPF, while the remaining six factors demonstrated a positive impact. These findings offer novel insights into the underlying etiopathological mechanisms associated with IPF.

## Introduction

Idiopathic pulmonary fibrosis (IPF) is a progressive lung disease featured by lung tissue scarring, with poor prognosis (1, 2). This condition is marked by the persistent development of scar tissue in the lungs, resulting in decreased lung flexibility, impaired oxygen exchange, and eventual respiratory failure and mortality (1, 3). Recent years have witnessed a global upsurge in the prevalence, hospitalization rate, and mortality associated with IPF, thus imposing a substantial burden on patients (4). However, due to the recognized unknown etiology, limited treatment options, and uncertain prognosis, it is imperative to conduct further investigations into the etiology and pathological mechanisms of IPF.

IPF is commonly believed to be influenced by the interplay of genetic and environmental factors, such as smoking, viral infections, occupational exposures to metal and wood chips, as well as agricultural activities (5). However, these risk factors fail to adequately account for the progressive nature of IPF. Numerous other factors may remain unidentified. An increasing number of studies have identified a higher prevalence of obesity, diabetes, cardiovascular disease in individuals with IPF (6–8). The occurrence of these three diseases is closely linked to abnormal endocrine and metabolic factors. However, previous studies on the effects of endocrine and metabolic factors on IPF have predominantly relied on observational research, yielding conflicting conclusions. The limitations of observational research, such as its inability to fully account for confounding factors, hinder its capacity to establish causal or reverse causality relationships. Regression models, which can only adjust for known confounders, are ineffective when confounders remain unidentified (9).

The Mendelian randomization (MR) method employs a valid instrumental variable (IV), including single nucleotide polymorphisms (SNPs), to simulate the random allocation of individual exposure and analyze the causal relationship between exposure and outcome (10). By randomly assigning the SNP allele during zygote formation prior to any exposure or outcome, it avoids associations with environmental confounders while minimizing the impact of reverse causality (9). Hence, the aim of this study is to employ MR to investigate the existence of a causal relationship between endocrine and metabolic factors and IPF.

## Methods

### Study design

The MR randomization method comprises three primary steps. The initial step involves evaluating whether the selected IV meets the three core assumptions. The second step encompasses conducting MR analysis to assess the causal effect between exposure and outcomes. Finally, the third step involves conducting sensitivity analysis to evaluate the reliability of the MR results.

### Data sources

To adhere to the fundamental principles of a two-sample MR design, data on exposure and outcome were collected from distinct European populations as described previously (11–13). The genome-wide association study (GWAS) datasets for 53 different exposures were extracted from sources such as the UK Biobank (UKBB) and the Genetic Investigation of ANthropometric Traits (GIANT), and can be accessed on the IEU OpenGWAS project website (https://gwas.mrcieu.ac.uk/). Furthermore, information on the IPF dataset was obtained from the study by Allen et al. (14). **Supplementary Table 1** provides comprehensive details on the GWAS datasets utilized in this study.

### Selection of IVs

The three fundamental assumptions are as follows: (1) IVs should be associated with the exposure; (2) IVs should not be associated with confounding factors; and (3) IVs should only be associated with the outcome through the exposure. To ensure adherence to these assumptions, specific implementation criteria were established. The inclusion criteria required a strong genetic association between the IVs and the exposure of interest, as indicated by a *P*-value < 5×10^-8^. Independent IVs with low levels of linkage disequilibrium (LD), characterized by an R^2^ value below 0.001, were identified using clumping methodology within a genomic window of 10 megabases. Following previous research findings, only IVs with minor allele frequencies (MAF) exceeding 0.01 were included in the analysis. Palindromic SNPs with ambiguous strand orientation were excluded from further analysis to ensure consistency in allele frequencies. F-statistics were calculated as measures of IV strength, with values greater than ten indicating minimal susceptibility to weak instrument bias (15).

### MR analysis

The primary method employed in this study is the inverse variance-weighted (IVW) method. Following the approach outlined in (16), the regression line intercept was constrained to cross zero. To assess the possible presence of unbalanced horizontal pleiotropy, we employed the weighted median (WM) and MR-Egger methods. The WM method remains unbiased as long as no more than 50% of the weight comes from invalid instruments. Unlike the IVW method, MR-Egger does not assume that the regression line intersects at zero. A non-zero intercept in MR-Egger indicates the presence of unbalanced horizontal pleiotropy. The slope of the regression line provides an estimate of the effect as explained by (17).

### Sensitivity analysis

To evaluate potential horizontal pleiotropy, we conducted an MR-Egger intercept test. In order to account for potential outliers, we incorporated pleiotropy-corrected data from MR-PRESSO. The Cochrane Q value was calculated to assess heterogeneity. To examine the influence of individual IVs on causal relationships and validate the reliability of the results, a leave-one-out sensitivity analysis was performed. In the MR analyses, causal effects were assessed using regression coefficients and odds ratios (ORs), along with their corresponding 95% confidence intervals (CIs), as the outcome variable was dichotomous. To address multiple comparisons, a false discovery rate (FDR) threshold of 5% was employed. All MR analyses were conducted using the TwoSampleMR package in R.

## Results

### Assessment of the IVs

In this study, MR analysis was utilized to examine the associations between endocrine and metabolic factors and IPF. The IVs for the traits displayed F-statistics ranging from 28.62 to 3093.37. These F-statistics indicate strong instrument strength, as detailed in **Supplementary Table 2**.

### Results of the MR analysis

**Figure 1:**
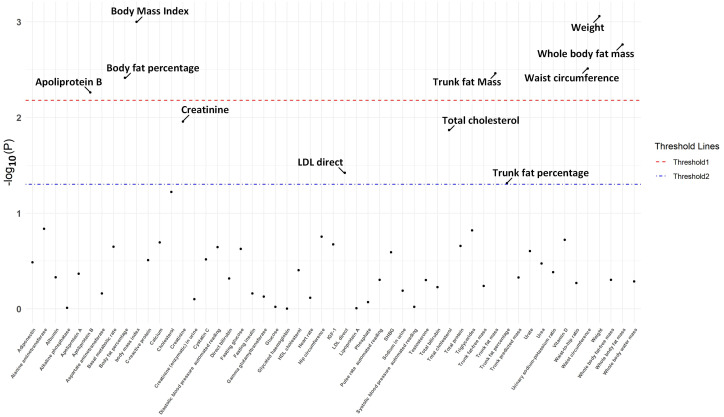
The *P*-value distribution of associations between endocrine and metabolic factors and idiopathic pulmonary fibrosis in the Mendelian randomization analysis. The significance threshold adjusted by False Discovery Rate is illustrated by the red dashed line, while the suggestive significance threshold, established at P = 0.05, is depicted by the blue dash-dotted line.

**Figure 2:**
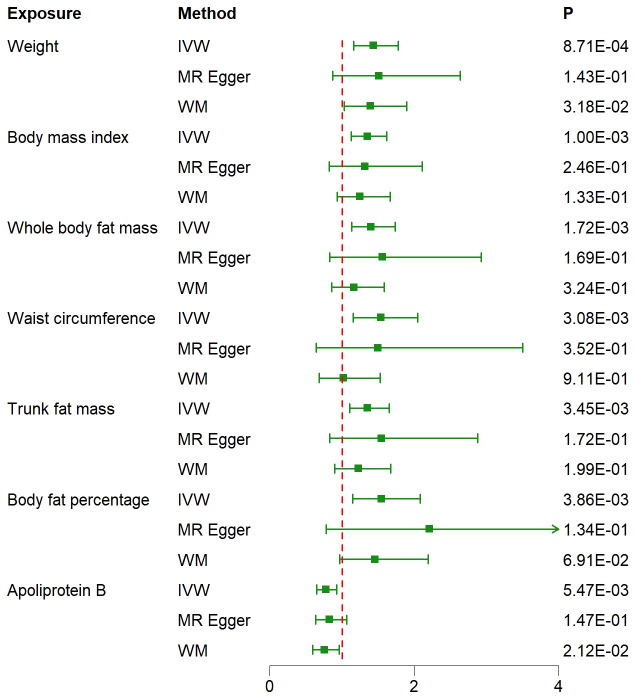
Associations between genetically predicted 7 traits and idiopathic pulmonary fibrosis examined by three MR methods. MR, Mendelian randomization; IVW, inverse-variance weighted; WM, weighted median; OR, odds ratio; CI, confidence interval.

**Figure 3:**
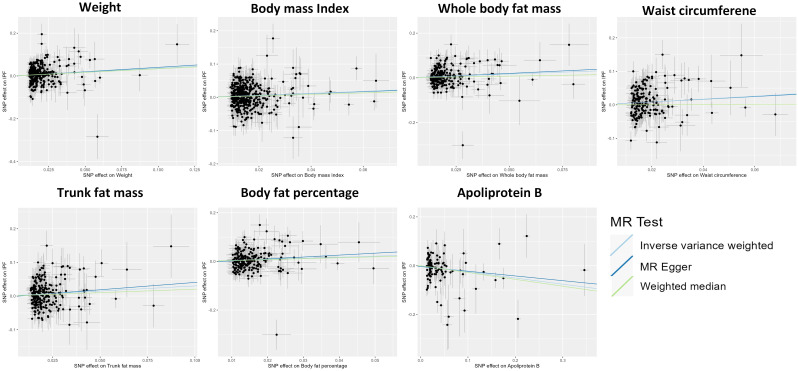
Scatter plot showing the causal effects of 7 traits on idiopathic pulmonary fibrosis. SNP, single nucleotide polymorphism.

The IVW approach in MR analysis revealed strong associations between genetically predicted traits and IPF. Specifically, the following factors showed significant links with IPF: “Weight” (OR= 1.44; 95% CI: 1.16, 1.78; *P*=8.71×10^-4^), “BMI” (OR= 1.35; 95% CI: 1.13, 1.62; *P*=1×10^-3^), “Whole body fat mass” (OR= 1.40; 95% CI: 1.14, 1.74; *P*=1.72×10^-3^), “Waist circumference (WC)” (OR= 1.54; 95% CI: 1.16, 2.05; *P*=3.08×10^-3^), “Trunk fat mass (TFM)” (OR=1.35; 95% CI: 1.10, 1.65; *P*=3.45×10^-3^), “Body fat percentage (BFP)” (OR= 1.55; 95% CI: 1.15, 2.08; *P*=3.86×10^-3^), “Apoliprotein B (ApoB)” (OR= 0.78; 95% CI: 0.65, 0.93; *P*=5.47×10^-3^). These findings, presented in **Figures 1** and **2** as well as **Supplementary Table 3**, remained statistically significant even after adjusting for multiple comparisons. **Figure 3** visually represents the causal relationships between the seven traits and IPF through a scatter plot.

### Results of the sensitivity analysis

**Figure 4:**
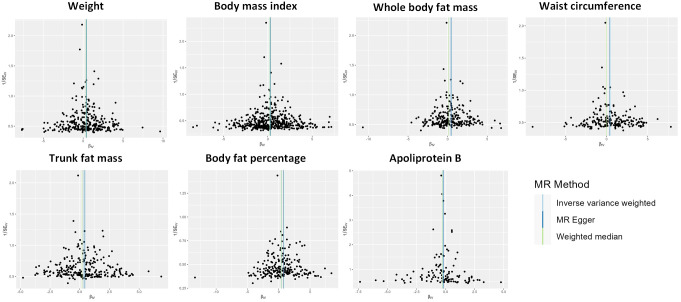
Funnel plot indicating the causal associations of 7 traits on idiopathic pulmonary fibrosis. SNP, single nucleotide polymorphism; IV, instrumental variable; SE, standard error.

The potential heterogeneity was tested (see **Figure 4** and **Supplementary Table 4**). The examination of the intercept term using the MR-Egger method did not reveal significant evidence of horizontal pleiotropy (see **Supplementary Table 5**). The findings from the MR-PRESSO analysis are consistent with the results mentioned earlier. It is worth noting that although MR-PRESSO identified outlier IVs, the results were not significantly altered (see **Supplementary Table 6**). As shown in **Supplementary Figure 1**, the leave-one-out analysis demonstrates that none of the individual SNPs were solely responsible for the observed outcomes. This sensitivity analysis method supports the reliability of our MR results.

In summary, our findings indicate a positive causal association between six traits (weight, BMI, whole body fat mass, WC, TFM, and BFP) and IPF. Additionally, a negative causal relationship between ApoB and IPF was also observed.

## Discussion

In this study, we conducted a two-sample Mendelian randomization analysis using summary statistics from the GWAS database. After conducting sensitivity analysis and correcting for multiple comparisons, we identified causal relationships between seven traits and IPF. Based on the various measurement methods employed, these seven traits will be discussed in the following three groups.

### The role of ApoB in IPF

The present study identified a negative causal relationship between ApoB and IPF. Previous studies on ApoB and lung fibrosis are limited, with most focusing on fibrosis in other organs such as the heart, liver, and kidney.

Cardiac fibrosis primarily refers to fibrotic changes in the blood vessels of the heart, contributing to the widespread occurrence of coronary atherosclerosis. This condition predominantly arises due to lipid deposition within the arterial intima, leading to the development of arterial intima fibrosis, subsequent formation of atheromatous plaques, and consequent obstruction of the coronary artery lumen. Low-density lipoprotein (LDL) cholesterol has long been recognized as the important protein implicated in the development of atherosclerosis (18). Nonetheless, emerging evidence from a recent study indicates that ApoB surpasses LDL cholesterol as a more effective predictor of cardiovascular risk (19). A cohort study involving 4232 participants revealed an association between elevated ApoB levels and the progression of atherosclerotic lesions (20). Furthermore, case-control studies comprising 57973 individuals demonstrated an association between ApoB truncation and a decreased incidence of coronary heart disease (21). ApoB serves as the primary apolipoprotein for triglyceride-rich lipoproteins [including milk fat particles and very low-density lipoprotein (VLDL)], LDL, medium-density lipoprotein, total cholesterol, and triglycerides (22). Apolipoprotein B exists primarily in two forms: Apolipoprotein-B100 (ApoB-100) and Apolipoprotein-B48 (ApoB-48) (23). ApoB-100 is the main protein responsible for transporting cholesterol (including VLDL and LDL) to tissues. Prior research has established the significant involvement of ApoB, specifically ApoB-100, in the advancement of atherosclerosis through its facilitation of atherogenic lipoprotein assembly (23). Moreover, ApoB-100 has been employed as a prognostic marker for steatosis, whereby heightened levels exhibit a substantial correlation with high-grade hepatic steatosis (24). The p210 vaccine, derived from the ApoB-100-related 20 amino acid peptide antigen, exhibited renal protection and decreased inflammation and fibrosis in CD8+ T cell-mediated apoE mice (25).

A previous study found that a decrease in ApoB in lung cancer leads to an increase in oxidative stress (26), and an increase in ApoB may presumably be related to a reduction in oxidative stress. IPF is a progressive, end-stage, age-related lung fibrosis disease, and its etiology is still unclear. Research has shown that mitochondrial ROS from epithelial cells, fibroblasts, and alveolar macrophages are involved in the development of IPF (27, 28). The endoplasmic reticulum (ER) is also a crucial contributor of ROS. Abnormal activation of ER stress response has been linked to the pathogenesis of IPF (29). Thus, an increase in ROS (which disrupts redox signaling and/or causes molecular damage) in mitochondria or ER contributes to the development of IPF (30). This study found that ApoB can decrease the risk of IPF, and it is hypothesized that the altered oxidative stress levels may play an important role in this process.

### The role of anthropometric measurements in IPF

Weight, BMI, and WC are commonly used anthropometric measures in clinical research (31–35). IPF consistently correlates with nutritional status, and anthropometry serves as an initial step in its evaluation (36). In our study, we established a positive causal relationship between weight, BMI, WC, and IPF. It is important to note that this relationship was specifically observed during the onset stage of the disease. However, in later stages of IPF, higher BMI and weight gain have been found to potentially mitigate adverse outcomes associated with the condition (37–40). Nevertheless, our results find support from another MR study, which demonstrates that each standard deviation increase in BMI is associated with a 40-70% higher risk of IPF (41). Another MR study reveals positive associations between BMI and WC with IPF risk. However, after correction for multiple comparisons, WC loses significance while BMI remains significant (5).

Limited studies have been conducted to investigate the mechanism by which anthropometric measurements impact IPF. IPF primarily focuses on the sensitivity of type II alveolar epithelial cells (AECII) to ER stress response (42–44). Previous studies have shown that ER stress leads to a decrease in PINK1 expression in AECIIs (45). This decrease in PINK1 expression results in enlarged mitochondria, decreased cellular viability, and activation of pro-fibrotic responses (45). Additionally, mitochondrial substances can induce the actions of cytokines in the lungs that possess proliferative and pro-fibrotic characteristics (45). WC is a simple measurement method that effectively determines abdominal fat distribution (46). Previous studies have found a correlation between increased WC, indicative of abdominal obesity, and reduced mitochondrial metabolism in muscles (47). In addition, significantly increased numbers of malformed and functionally impaired mitochondria have been observed in AECIIs in the lungs of IPF patients (45). Interestingly, our study revealed a positive causal relationship between WC and IPF. Therefore, we hypothesize that decreased mitochondrial metabolism in AECIIs may interplay with the accumulation of abdominal obesity, leading to an increase in IPF.

### The role of body composition in IPF

This study uncovered a significant positive causal relationship between whole body fat mass, TFM, BFP, and IPF. Whole body fat mass, TFM, and BFP are widely recognized as reliable indicators of body composition. Assessing body composition ranks as the second most crucial factor in evaluating the physical nutritional status of IPF patients (36). However, there is a dearth of research on the link between body composition and IPF, with one study reporting that each standard deviation increase in BFP level raises the risk of IPF by approximately 40-70% (41), which aligns with our findings. The underlying mechanism by which whole body fat mass, TFM, and BFP contribute to IPF remains unknown and requires further investigation in future studies.

## Strengths and limitations

Our study possesses numerous strengths. Firstly, MR studies offer various advantages compared to observational studies. By utilizing genetic variation as an IV, this study effectively mitigates the influence of confounding factors and reverse causality. Secondly, we conducted a novel investigation into the causal relationship between multiple endocrine and metabolic factors and IPF. Additionally, we performed several sensitivity analyses to reinforce the reliability of our findings.

Our study is subject to several limitations. Firstly, our study was conducted exclusively on populations of European ancestry, which limits the generalizability of our findings to other populations. Therefore, caution should be exercised when extrapolating the results to populations of different ancestries. Secondly, our study can only establish causality based on adherence to linear associations, and we cannot explicitly confirm that the relationship between these endocrine and metabolic factors and IPF follows strict linear causality. For example, a cohort study conducted in the UK identified a J-shaped association between BMI and respiratory disease-related mortality (48). Lastly, while efforts were made to minimize selective bias, its complete elimination may not have been achieved. Additionally, due to the absence of certain individual baseline data in the GWAS, we were unable to stratify the analyses by covariates of interest, such as age and gender.

## Conclusion

This study found causal associations between seven traits and IPF. Specifically, ApoB had a negative impact on IPF, whereas the other six factors had positive effects. These findings provide novel insights into the underlying etiopathological mechanism of IPF. However, the specific mechanism by which these factors affect IPF remains unknown and warrants further investigation.

## Data availability statement

The original contributions presented in the study are included in the article/**Supplementary Material**. Further inquiries can be directed to the corresponding authors.

## Ethics statement

The GWAS included in this work were approved by their relevant review board, and informed consent were given by all participants.

## Author contributions

YJ: Writing – original draft. RC: Writing – original draft. SX: Writing – original draft. YD: Writing – original draft. MZ: Writing – original draft. MB: Writing – review & editing. BH: Writing – review & editing. SL: Writing – review & editing.

## Supplementary material

Supplementary Table 1 Information of GWAS summary datasets used in MR analyses. GWAS, genome-wide association study; ID, Identification; IPF, Idiopathic Pulmonary Fibrosis; MR, Mendelian randomization; ukb, UK Biobank; HDL, High-density lipoprotein; IGF-1, Insulin-like growth factor 1; LDL, Low-density lipoprotein; SHBG, Sex hormone-binding globulin.

Supplementary Table 2 The F-statistics of IVs. IVs, instrumental variables; SNP, single nucleotide polymorphism.

Supplementary Table 3 MR analysis results. MR, Mendelian randomization; SNP, single nucleotide polymorphism; OR, Odds ratio; CI, confidence interval; IVW, inverse-variance weighted; WM, Weighted median.

Supplementary Table 4 Heterogeneity test results.

Supplementary Table 5 The pleiotropy test results. SE, standard error.

Supplementary Table 6 MR-PRESSO analyses between exposures and outcomes with outlier. MR, Mendelian randomization; SD, standard deviation.
