# Supplementary material for: Endocrine and metabolic factors and the risk of idiopathic pulmonary fibrosis: a Mendelian randomization study
Source: Front Endocrinol (Lausanne). 2024 Jan 8;14:1321576. doi: 10.3389/fendo.2023.1321576 (PMC10801027; doi:10.3389/fendo.2023.1321576)
Supplement: Supplementary Table 1 — Information of GWAS summary datasets used in MR analyses. GWAS, genome-wide association study; ID, Identification; IPF, Idiopathic Pulmonary Fibrosis; MR, Mendelian randomization; ukb, UK Biobank; HDL, High-density lipoprotein; IGF-1, Insulin-like growth factor 1; LDL, Low-density lipoprotein; SHBG, Sex hormone-binding globulin. [file DataSheet_1.docx]

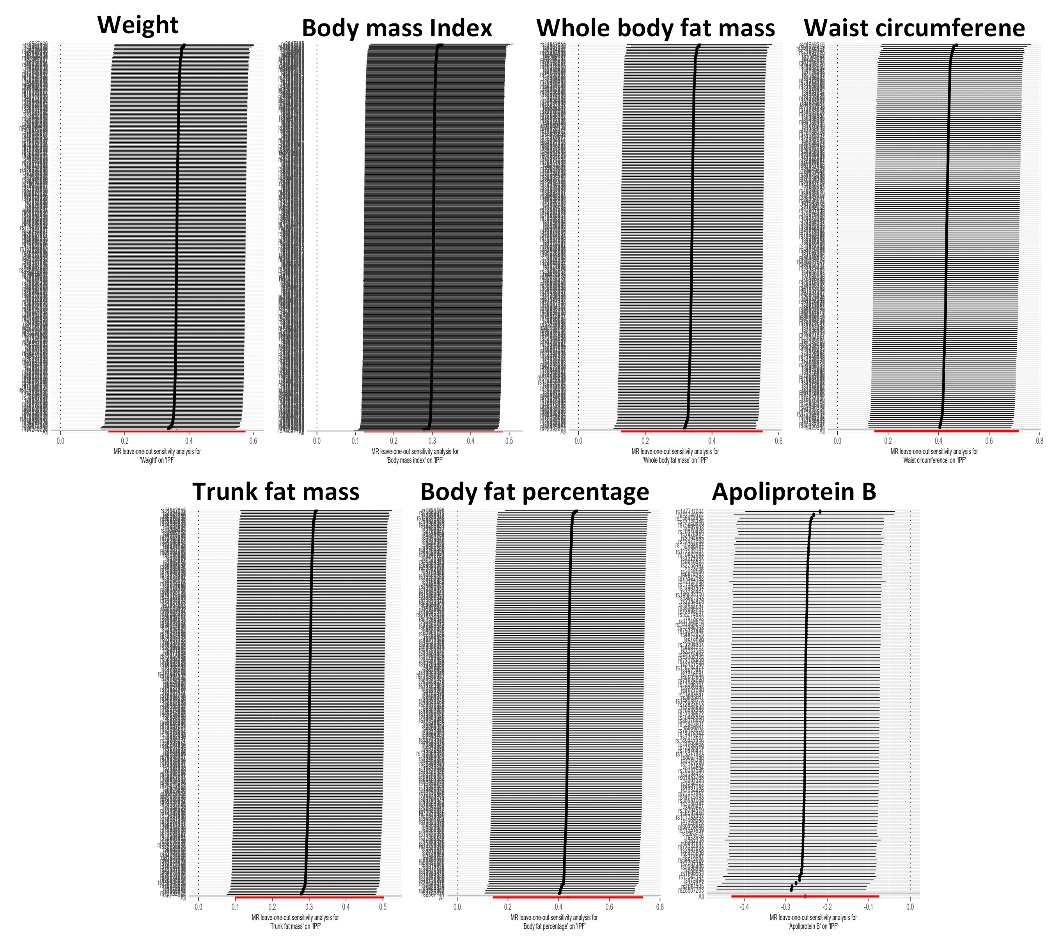


Supplementary Figure 1. Leave-one-out sensitivity analysis examining the causal estimates of 7 traits on idiopathic pulmonary fibrosis by the IVW method after exclude a specific SNP from the analysis. The red line represents the IVW estimate of all SNPs on each outcome. MR: Mendelian randomization; SNP: single nucleotide polymorphism; IVW: inverse-variance

**Supplementary Table 1**. Information of GWAS summary datasets used in MR analyses

| Traits | GWAS.ID |
| --- | --- |
| IPF | Allen.et.al |
| Adiponectin | ieu-a-1 |
| Alanine aminotransferase | ukb-d-30620 |
| Albumin | ukb-d-30600 |
| Alkaline phosphatase | ukb-d-30610 |
| Apoliprotein A | ukb-d-30630 |
| Apoliprotein B | ukb-d-30640 |
| Aspartate aminotransferase | ukb-d-30650 |
| Basal metabolic rate | ukb-a-268 |
| Body fat percentage | ukb-a-264 |
| Body mass index | ieu-b-40 |
| Calcium | ukb-d-30680 |
| Cholesterol | ukb-d-30690 |
| C-reactive protein | ukb-d-30710 |
| Creatinine | ukb-d-30700 |
| Creatinine (enzymatic) in urine | ukb-a-333 |
| Cystatin C | ukb-d-30720 |
| Diastolic blood pressure automated reading | ukb-a-359 |
| Direct bilirubin | ukb-d-30660 |
| Fasting glucose | ieu-b-114 |
| Fasting insulin | ieu-b-116 |
| Gamma glutamyltransferase | ukb-d-30730 |
| Glucose | ukb-d-30740 |
| Glycated haemoglobin | ukb-d-30750 |
| HDL cholesterol | ukb-d-30760 |
| Heart rate | ieu-a-1056 |
| Hip circumference | ukb-a-388 |
| IGF-1 | ukb-d-30770 |
| LDL direct | ukb-d-30780 |
| Lipoprotein A | ukb-d-30790 |
| Phosphate | ukb-d-30810 |
| Pulse rate automated reading | ukb-a-3 |
| SHBG | ukb-d-30830 |
| Sodium in urine | ukb-a-335 |
| Systolic blood pressure automated reading | ukb-a-360 |
| Testosterone | ukb-d-30850 |
| Total bilirubin | ukb-d-30840 |
| Total cholesterol | ieu-a-301 |
| Total protein | ukb-d-30860 |
| Triglycerides | ukb-d-30870 |
| Trunk fat mass | ukb-a-291 |
| Trunk fat percentage | ukb-a-290 |
| Trunk fat-free mass | ukb-a-292 |
| Trunk predicted mass | ukb-a-293 |
| Urate | ukb-d-30880 |
| Urea | ukb-d-30670 |
| Urinary sodium-potassium ratio | ieu-b-72 |
| Vitamin D | ukb-d-30890 |
| Waist circumference | ukb-a-382 |
| Waist-to-hip ratio | ieu-a-72 |
| Weight | ukb-a-249 |
| Whole body fat mass | ukb-a-265 |
| Whole body fat-free mass | ukb-a-266 |
| Whole body water mass | ukb-a-267 |
| Abbreviation: GWAS: genome-wide association study; ID: Identification; IPF: Idiopathic Pulmonary Fibrosis; MR: Mendelian randomization; ukb: UK Biobank; HDL: High-density lipoprotein; IGF-1: Insulin-like growth factor 1; LDL: Low-density lipoprotein; SHBG: Sex hormone-binding globulin. | |

**Supplementary Table 2**. The F-statistics of IVs

| Exposure | Number of SNPs used as IVs | F-statistic | |
| --- | --- | --- | --- |
|  |  | Min | Max |
| Weight | 291 | 29.73 | 772.58 |
| Body mass index | 442 | 28.62 | 1426.17 |
| Whole body fat mass | 248 | 29.75 | 636.20 |
| Waist circumference | 195 | 29.76 | 660.76 |
| Trunk fat mass | 248 | 29.79 | 546.41 |
| Body fat percentage | 219 | 30.01 | 433.59 |
| Apoliprotein B | 115 | 29.82 | 3093.37 |
| Abbreviation: IVs: instrumental variables; SNP: single nucleotide polymorphism. | | | |

**Supplementary Table 3.** MR analysis results

| Expoure | Method | Number of SNPs | OR | 95% CI | *P* |
| --- | --- | --- | --- | --- | --- |
| Weight | IVW | 291 | 1.44 | 1.16~1.78 | 8.71E-04 |
| Weight | MR Egger | 291 | 1.51 | 0.87~2.63 | 1.43E-01 |
| Weight | WM | 291 | 1.40 | 1.03~1.89 | 3.18E-02 |
| Body mass index | IVW | 442 | 1.35 | 1.13~1.62 | 1.00E-03 |
| Body mass index | MR Egger | 442 | 1.32 | 0.83~2.11 | 2.46E-01 |
| Body mass index | WM | 442 | 1.25 | 0.93~1.67 | 1.33E-01 |
| Whole body fat mass | IVW | 248 | 1.40 | 1.14~1.74 | 1.72E-03 |
| Whole body fat mass | MR Egger | 248 | 1.56 | 0.83~2.93 | 1.69E-01 |
| Whole body fat mass | WM | 248 | 1.17 | 0.86~1.58 | 3.24E-01 |
| Waist circumference | IVW | 195 | 1.54 | 1.16~2.05 | 3.08E-03 |
| Waist circumference | MR Egger | 195 | 1.50 | 0.64~3.50 | 3.52E-01 |
| Waist circumference | WM | 195 | 1.02 | 0.69~1.53 | 9.11E-01 |
| Trunk fat mass | IVW | 248 | 1.35 | 1.10~1.65 | 3.45E-03 |
| Trunk fat mass | MR Egger | 248 | 1.55 | 0.83~2.88 | 1.72E-01 |
| Trunk fat mass | WM | 248 | 1.23 | 0.90~1.68 | 1.99E-01 |
| Body fat percentage | IVW | 219 | 1.55 | 1.15~2.08 | 3.86E-03 |
| Body fat percentage | MR Egger | 219 | 2.21 | 0.79~6.24 | 1.34E-01 |
| Body fat percentage | WM | 219 | 1.46 | 0.97~2.19 | 6.91E-02 |
| Apoliprotein B | IVW | 115 | 0.78 | 0.65~0.93 | 5.47E-03 |
| Apoliprotein B | MR Egger | 115 | 0.82 | 0.64~1.07 | 1.47E-01 |
| Apoliprotein B | WM | 115 | 0.76 | 0.60~0.96 | 2.12E-02 |
| Abbreviation: MR: Mendelian randomization; SNP: single nucleotide polymorphism; OR:Odds ratio; CI: confidence interval; IVW: inverse-variance weighted; WM: Weighted median. | | | | | |

**Supplementary Table 4.** Heterogeneity test results

| Exposure | Q | Q_df | Q_*P* |
| --- | --- | --- | --- |
| Weight | 382.90 | 290 | 2.03E-04 |
| Body mass index | 464.07 | 441 | 2.16E-01 |
| Whole body fat mass | 301.41 | 247 | 1.02E-02 |
| Waist circumference | 270.13 | 194 | 2.47E-04 |
| Trunk fat mass | 289.84 | 247 | 3.17E-02 |
| Body fat percentage | 263.71 | 218 | 1.86E-02 |
| Apoliprotein B | 154.21 | 114 | 7.24E-03 |

**Supplementary Table** **5**. The pleiotropy test results

| Exposure | Egger intercept | *SE* | *P* |
| --- | --- | --- | --- |
| Weight | -0.0011 | 0.0055 | 8.40E-01 |
| Body mass index | 0.0004 | 0.0039 | 9.08E-01 |
| Whole body fat mass | -0.0022 | 0.0065 | 7.31E-01 |
| Waist circumference | 0.0005 | 0.0079 | 9.46E-01 |
| Trunk fat mass | -0.0030 | 0.0067 | 6.55E-01 |
| Body fat percentage | -0.0057 | 0.0081 | 4.81E-01 |
| Apoliprotein B | -0.0034 | 0.0055 | 5.39E-01 |
| Abbreviation:SE: standard error. | | | |

**Supplementary Table 6.** MR-PRESSO analyses between exposures and outcomes with outlier

| Exposure | MR Analysis | Causal Estimate | SD | *P*-value |
| --- | --- | --- | --- | --- |
| Weight | Outlier-corrected | 0.36 | 0.11 | 6.61E-04 |
| Whole body fat mass | Outlier-corrected | 0.36 | 0.10 | 6.09E-04 |
| Waist circumference | Outlier-corrected | 0.46 | 0.14 | 1.44E-03 |
| Body fat percentage | Outlier-corrected | 0.47 | 0.14 | 1.27E-03 |
| Apoliprotein B | Outlier-corrected | -0.24 | 0.09 | 7.22E-03 |
| Abbreviation: MR: Mendelian randomization; SD: standard deviation. | | | | |
